# Fetal growth restriction is a host specific response to infection with an impaired spiral artery remodeling-inducing strain of *Porphyromonas gingivalis*

**DOI:** 10.1038/s41598-020-71762-9

**Published:** 2020-09-03

**Authors:** Tanvi Tavarna, Priscilla L. Phillips, Xiao-jun Wu, Leticia Reyes

**Affiliations:** 1 Department of Pathobiological Sciences, University of Wisconsin - Madison, School of Veterinary Medicine, 2015 Linden Drive, Madison, WI 53706 USA; 2 Microbiology and Immunology, Kirksville College of Osteopathic Medicine, A.T. Still University of Health Sciences, Kirksville, MO USA

**Keywords:** Microbiology, Diseases, Intrauterine growth, Angiogenesis

## Abstract

*Porphyromonas gingivalis* is a periodontal pathogen implicated in a range of pregnancy disorders that involve impaired spiral artery remodeling (ISAR) with or without fetal growth restriction (FGR). Using a rodent periodontitis model, we assessed the ability of *P. gingivalis* to produce ISAR and FGR in Sprague Dawley (SD) and Wistar (WIS) rats. Both infected SD and WIS rats developed ISAR, but only WIS rats developed FGR despite both rat strains having equivalent microbial loads within the placenta. Neither maternal systemic inflammation nor placental (fetal) inflammation was a feature of FGR in WIS rats. Unique to infected WIS rats, was loss of trophoblast cell density within the junctional zone of the placenta that was not present in SD tissues. In addition, infected WIS rats had a higher proportion of junctional zone trophoblast cells positive for cytoplasmic high temperature requirement A1 (Htra1), a marker of cellular oxidative stress. Our results show a novel phenomenon present in *P. gingivalis-*induced FGR, with relevance to human disease since dysregulation of placental Htra1 and placental oxidative stress are features of preeclamptic placentas and preeclampsia with FGR.

## Introduction

*Porphyromonas gingivalis,* a Gram-negative bacterial pathogen, is one of the causative agents of generalized periodontitis in humans, which involves damage to the tooth-supporting structures^1^. *P. gingivalis* is also implicated in a variety of pregnancy complications including recurrent spontaneous abortion^2^, preterm labor^3,4^, and preeclampsia with or without fetal growth restriction (FGR)^5–8^. Similarly, experimental infection with *P. gingivalis* may lead to spontaneous preterm birth^9,10^, fetal death^11,12^, and/or fetal growth restriction^13–15^ in rodents.

Impaired spiral artery remodeling (ISAR) occurs when the normal physiologic process that converts high resistance uterine arteries into dilated, low resistance vessels is disrupted during pregnancy^16^. ISAR has been detected in women suffering from recurrent spontaneous abortion, preterm labor, preeclampsia, and/or FGR^17–19^; the same types of complications that have been linked to *P. gingivalis* infection^2–8^. We previously provided proof of concept that *P. gingivalis* infection produces ISAR in Sprague Dawley (SD) rats^11^. Surprisingly, FGR was not observed in our model. Since *P. gingivalis*-induced FGR is linked to overexpression of pro-inflammatory cytokines within the placenta^13–15^ and has been seen in studies that used Wistar (WIS) rats^10,15^, we hypothesized that the host immune response to infection is a determinant in whether or not FGR occurs.

FGR refers to the failure of the developing fetus to achieve optimal genetically determined growth in utero* and* is an important cause of fetal and neonatal morbidity and mortality^20,21^. FGR is multifactorial and can be broadly categorized into maternal, fetal, and placental in origin. Some examples of maternal causes of FGR include underlying vascular disease, immune mediated diseases, substance abuse or toxicities, infectious diseases, genetic disorders, and multiple pregnancies^20^. FGR can also be caused by utero-placental factors such as placental infection, and poor implantation or inadequate placentation that can be an extension of maternal vascular disease including ISAR^22^. Less common causes of FGR include fetal malformations, inborn errors of metabolism, and chromosomal abnormalities^21^.

To test our hypothesis, we infected both SD and WIS rats with *P. gingivalis* using a periodontitis model of infection^23^, and pregnant dams were assessed for the development of ISAR and FGR. Herein we report that *P. gingivalis* infection induced ISAR in both rat strains, but only infected WIS dams exhibited FGR. In contrast to other studies^10,15^, neither maternal systemic inflammation nor placental (fetal) inflammation was a feature of FGR. In addition, placental microbial load was equivalent in both rat strains suggesting this was not a determining factor in the development of FGR. However, we found structural changes within the junctional zone of the placenta of infected WIS rats that was not present in SD placentas. Furthermore, a higher proportion of junctional zone trophoblast cells in infected WIS rats were positive for cytoplasmic high temperature requirement A1 (Htra1). Cytoplasmic expression of Htra1 is a marker of cellular oxidative stress^24,25^, and placental oxidative stress secondary to tissue hypoxia is a feature of growth restricted placentas^26^. Notably, dysregulation of placental Htra1 expression occurs in preeclamptic placentas with or without FGR^27,28^. Our results show a novel phenomenon whereby *P. gingivalis* promotes FGR, which may have clinical relevance to human disease.

## Results

### *P. gingivalis* disrupts uterine spiral artery remodeling in both rat strains.

**Figure 1:**
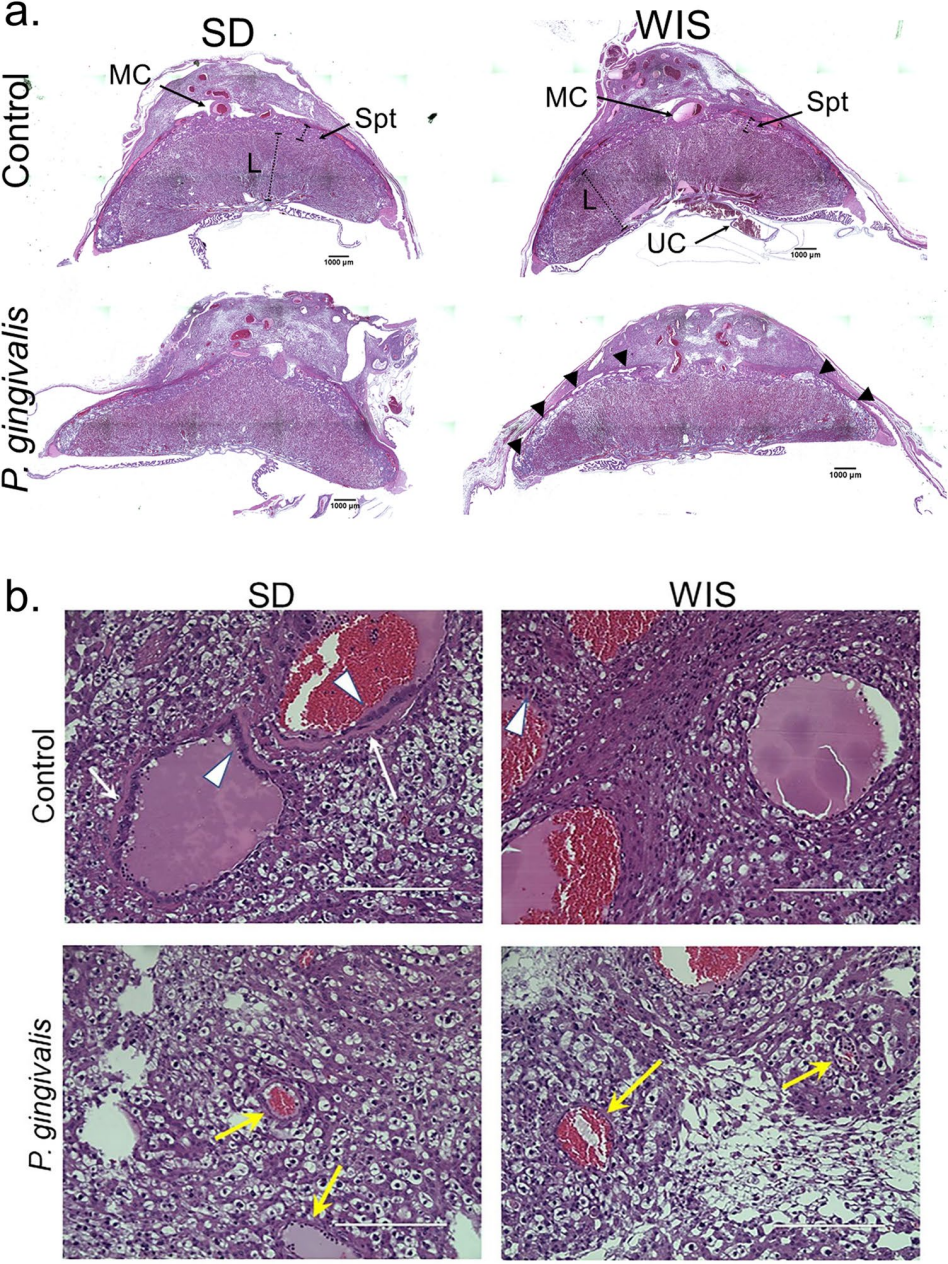
(**a**) Representative images of GD18 SD and WIS utero-placenta from control and *P. gingivalis* infected dams. Images are tiled composites of individual pictures taken at ×4 magnification with an EVOS Auto FL imaging system. Scale bar = 1,000 µm. MC = main channel; L = labyrinth; Spt = spongiotrophoblasts within the junctional zone; UC = umbilical cord; black arrowheads mark the edge of the placenta (basal plate) in contact with the decidua. (**b**) Representative images of remodeled (control SD and WIS) and poorly remodeled spiral arteries (*P. gingivalis* SD and WIS). White arrows indicate fibrinoid deposition. White arrowheads indicate EVT. Yellow arrows indicate poorly remodeled vessels. Scale bar = 400 µm. Composite image was created with Adobe Photoshop 2020 v. 21.1.3 (www.adobe.com).

**Figure 2:**
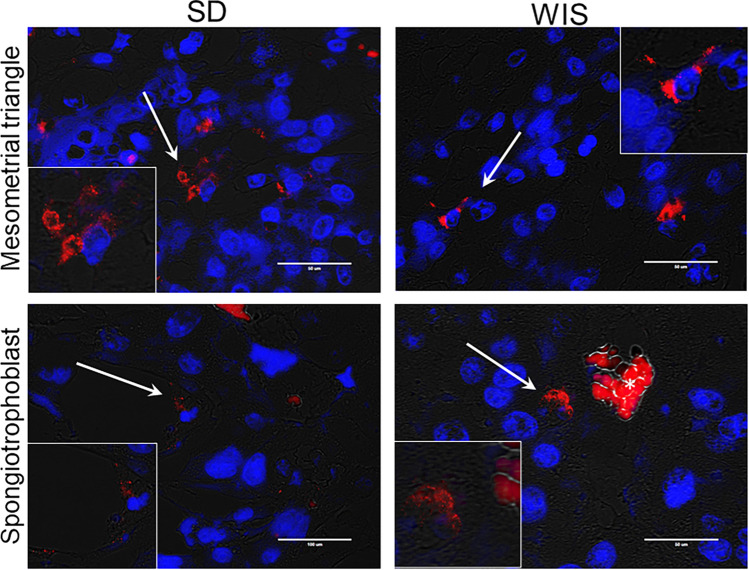
Detection of *P. gingivalis* in the mesometrial triangle and the junctional zone of the placenta of SD and WIS rats. Representative images of positive specimens. White arrows point to *P. gingivalis* (red) that is magnified in the corresponding inset. Transillumination (grey) was used to define the tissue architecture. Nuclei were stained with DAPI (blue). Scale bar = 50 µm. Asterisk in the bottom right panel indicates autofluorescent maternal red blood cells. Stain controls available in Supplement file Fig. S2. Composite image was created with Adobe Photoshop 2020 v. 21.1.3 (www.adobe.com).

Adequately remodeled spiral arteries display large lumen diameters with loss of the surrounding smooth muscle layer, fibrinoid deposition within the vessel wall, and infiltration by fetal derived extravillous trophoblasts (EVT)^29^. Since we previously established that *P. gingivalis* infection impairs uterine spiral artery remodeling in pregnant SD rats^11^, we first determined if the same occurs in WIS dams. Formalin fixed utero-placental specimens from control and infected rats were selected for morphometric analysis based on the quality of the section (i.e., confirmation that it traversed the center of the utero-placental unit and that the entire uterus, placenta were intact, as shown in Fig. 1a. The in situ presence of *P. gingivalis* within the uteroplacental tissues of inoculated dams was confirmed by immunofluorescent histology (Fig. 2).

**Figure 3:**
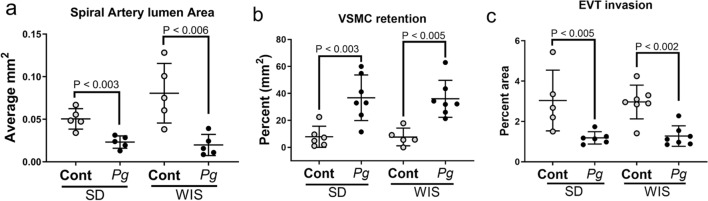
The impact of *P. gingivalis* infection on spiral artery remodeling in SD and WIS rats measured as reduced spiral artery lumen size, retention of VSMC in spiral arterioles, and decreased extravillous trophoblast (EVT) invasion into mesometrium. (**a**) Came from uteroplacental specimens that were fixed *in toto* to preserve uterine vascular architecture. (**b**) and (**c**) include utero-placental specimens used in panel **a** as well as specimens in which the fetus was removed from the uteroplacenta before fixation. Horizontal lines indicate mean ± standard deviation. Data within each rat strain was analyzed by student’s t test. Composite image was created with Adobe Photoshop 2020 v. 21.1.3 (www.adobe.com).

By gestation day 18, the extent of spiral artery remodeling among control SD and WIS rats was equivalent (*P* > 0.12 for all analyses). Infection with *P. gingivalis* reduced spiral arterial lumen area (Fig. 3a), increased retention of vascular smooth muscle cells (VSMCs) (Fig. 3b, supplement file, Fig. S3), and decreased EVT invasion into the mesometrium (Fig. 3c, supplement file, Fig. S3) of both rat strains. *P. gingivalis* was identified within the mesometrial stroma surrounding spiral arteries as well as scattered through the stromal tissue in both rat strains (Fig. 2). The similar effect of infection in both rat strains indicates that ISAR is a pathogen-induced event rather than a host-specific response.

### *P. gingivalis*-infection did not alter maternal serum cytokine/chemokine concentrations

We profiled serum cytokine/chemokine levels in SD and WIS dams since experimental infection with *P. gingivalis* has been shown to increase maternal pro-inflammatory responses^10,13,15^. Neither TNF-α, GM-CSF, IL-12p70, nor IL-1α were detected in either rat strain (data not shown). Among the detected cytokines/chemokines that were assayed, *P. gingivalis* infection did not alter serum maternal cytokine/chemokine concentrations in either rat strain relative to their controls (Supplement file, Fig. S4).

### Only fetuses from *P. gingivalis*-infected WIS dams exhibit growth restriction

*P. gingivalis* infection did not have an adverse effect on fertility rate nor gestation length. Only 1 control SD rat failed to get pregnant after confirmation of successful breeding. In WIS rats, one control animal and one infected animal failed to become pregnant despite successful breeding. None of the pregnant rats in any of the treatment groups went into spontaneous labor before termination of the study (gestation day 18).

The impact of *P. gingivalis* infection on fetal heath was evaluated by litter size, the number of fetal resorptions (deaths) per litter, and fetal weight. Maternal infection did not affect litter size in either rat strain. In SD rats, mean litter size ± SD was 9 ± 4 in controls (n = 10) and 12 ± 3 in infected animals (n = 8) (*P* = 0.1161). In WIS rats, mean litter size ± SD was 11 ± 3 in controls (n = 7) versus 12 ± 3 in infected animals (n = 10) (*P* = 0.2304).

**Figure 4:**
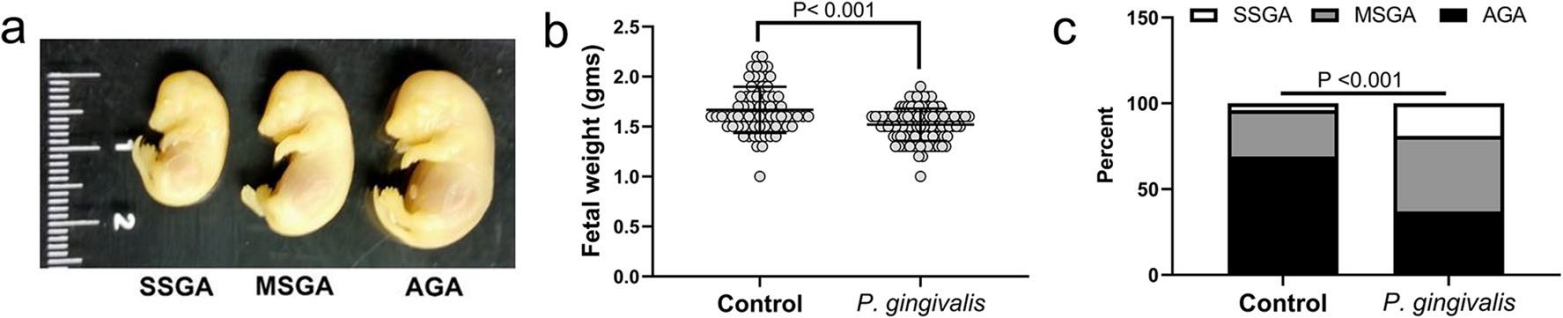
*P. gingivalis* infection induces FGR in WIS rats. (**a**) Representations of severely small for gestational age (SSGA), moderately small for gestational age (MSGA) and average for gestational age (AGA) fetuses. (**b**) Fetal weights (g) from 7 control and 10 infected dams. Data was analyzed by student’s t test. (**c**) Proportion of SSGA, MSGA and AGA fetuses in litter size matched control and infected dams. Data was analyzed by chi square test (n = 74 controls and 84 infected). Composite image was created with Adobe Photoshop 2020 v. 21.1.3 (www.adobe.com).

Infection did not affect the number of fetal resorptions in either rat strain. In SD dams, the proportion of fetal resorptions per litter was 9.3 ± 10% in controls (n = 10) versus 12 ± 14% in *P. gingivalis* infected animals (n = 8) (P = 0.6195). In WIS rats, the rate of fetal resorptions per litter was 11.4 ± 17% in controls (n = 7) versus 12 ± 17% in *P. gingivalis* infected animals (n = 10) (*P* = 0.9467). In contrast, fetal growth rates were different among both rat strains. In uninfected control groups, SD rats had smaller fetuses than gestational age matched WIS dams; the mean weight (g) ± standard deviation in the SD group was 1.53 ± 0.17 versus 1.67 ± 0.23 in the WIS group (*P* < 0.001). Similar to our previous report^11^, infection with *P. gingivalis* did not affect fetal growth in SD dams, which was 1.54 ± 0.17 g in the infected group (*P* = 0.6164). In contrast, fetuses from *P. gingivalis* infected WIS dams were significantly smaller than their control counterparts (Fig. 4a,b, *P* < 0.001).

To further define the extent of FGR in WIS rats, we modified FGR criteria used in clinical medicine^30^ to the rat model. Specifically, fetuses were classified as severely small for gestational age (SSGA) if their weight was less than the 10th percentile of control animals, which was < 1.3 g body weight. Fetuses were classified as moderately small for gestational age (MSGA) if their weight was between the 10th and 33rd percentile of control animals, which ranged from 1.3 to < 1.6 g. Fetuses were considered average for gestational age (AGA) if they weighed ≥ 1.6 g. Because litter size can have an inverse effect on fetal weight^31^, only litters with equivalent numbers between control and infected animals were included in this analysis. This resulted in the exclusion of one infected dam that had 17 fetuses. Based on this criteria, *P. gingivalis* infected dams had a greater proportion of both SSGA [4% in control (n = 3) versus 17% in infected (n = 17)] and MSGA [27% in control (n = 20) versus 37% in infected (n = 39)] fetuses compared to controls (Fig. 4c, *P* < 0.001). Based on detection of the *Sry* gene in DNA extracts from *P. gingivalis* infected placenta*,* FGR in infected WIS placenta was not linked to fetal sex since the mean weight in females was 1.50 ± 0.09 g versus 1.52 ± 0.1 g in males (*P* = 0.7128, n = 12).

Since FGR can be a consequence of poor maternal nutrition^32^, we monitored maternal weight gain in control and infected dams as an indirect measure of nutritional status (Supplement file, Fig. S5). We found no difference in the rate of weight gain among control and infected SD and WIS dams suggesting poor nutrition was not a likely factor for FGR in WIS rats.

We next assessed the microbial load and the in situ location of bacteria within the placenta since this could be a contributing factor in FGR^13,14^. Microbial load in placental tissues used for gene expression analysis was equivalent based on qPCR. In SD placenta, the mean ± SD Cq was 36.44 ± 0.79 (approximately 0.00015 ± 0.0001 pg/ng of DNA) versus 36.49 ± 1.5 Cq (0.00019 ± 0.00003 pg/ng of DNA) in WIS placenta (n = 6, *P* > 0.61). The location of *P. gingivalis* within the placenta was readily detected within the junctional zone in both rat strains (Fig. 2). Specifically, bacterial aggregates were found in association with spongiotrophoblasts and glycogen cells in both rat strains. Bacteria were not detected in the chorioamnion, yolk sac or within the amniotic cavity in either rat strain (data not shown). Taken together, our results suggest that microbial load is not a major determinant of FGR in our model of infection.

### Placentitis is not a feature of *P. gingivalis*-induced FGR

FGR can involve chronic inflammatory lesions of the placenta characterized by infiltration of the tissue by lymphocytes, plasma cells, and/or macrophages^30,33^. FGR placentas that involve vascular pathology may exhibit an increase in degenerative changes that are normally found in aging placentas such as hemorrhage, increased fibrinoid deposition, infarcts, and a band-like distribution of coagulative necrosis at the choriodecidual interface^34,35^. Uteroplacental specimens used for spiral artery measurements underwent histologic evaluation with the reader blinded to treatment.

The extent of inflammatory infiltrates into the placenta never exceeded 20% of the entire tissue section in both SD and WIS control and infected groups. Mononuclear cell infiltrates were mainly observed within the choriodecidual junction (Supplement file Fig. S6). The proportion of placental specimens with the most extensive involvement, which was 10–20% of the tissue section, were also similar among the groups: 1 of 7 in control SD, 2 of 7 in infected SD (*P* > 0.9999 by Fisher’s exact test), 2 of 7 in control WIS, and 3 of 7 in infected WIS (*P* > 0.9999 by Fisher’s exact test).

**Figure 5:**
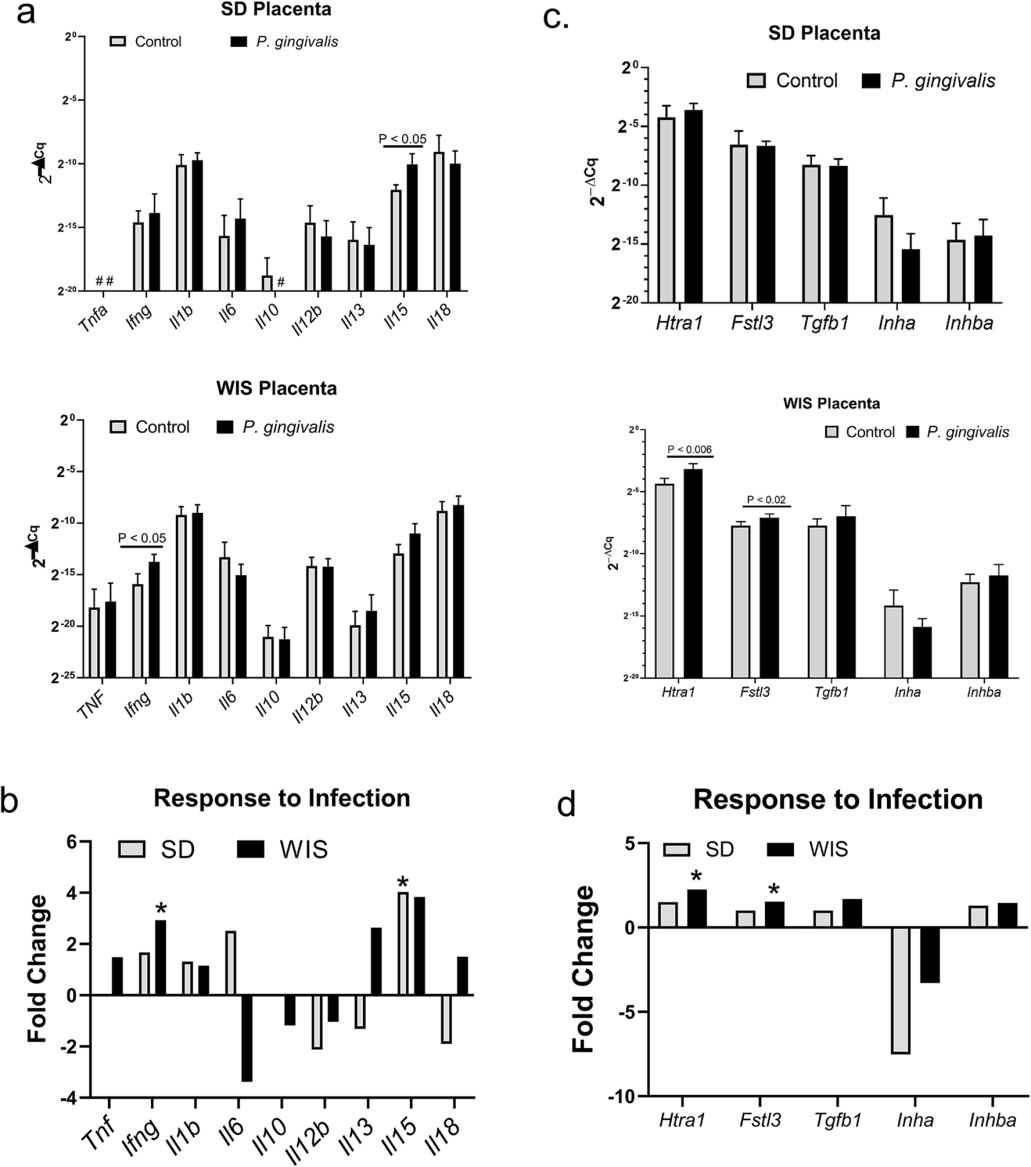
Placental gene expression profiles of SD and WIS rats. **a** Cytokine and chemokine gene expression measured by RT-qPCR. Values are the mean 2^−ΔCq^ ± SD determined by the comparative Cq method^40^ using *Actb* as the reference gene (n = 6). ^#^Indicates genes that were not detected by RT-qPCR in SD rats. (**b**) The mean fold response of the same cytokine and chemokine genes shown in (**a**). Values were generated by dividing the mean 2^−ΔCq^ (n = 6) of the infected group by the mean 2^−ΔCq^ (n = 6) of the corresponding control group. *Indicates the 2^−ΔCq^ values that were significantly affected by infection in panel (**a**) based on student’s t test. (**c**) Expression of genes involved in placental morphogenesis measured by RT-qPCR. Values are the mean 2^−ΔCq^ ± SD determined by the comparative Cq method^40^ with the corresponding *Actb* used as the reference gene (n = 6). (**d**) The mean fold response of the same placental genes shown in panel **c**. *Indicates the 2^−ΔCq^ values that were significantly affected by infection in panel **c** based on Student’s t test. Composite image was created with Adobe Photoshop 2020 v. 21.1.3 (www.adobe.com).

We next examined a select set of cytokine and chemokine genes including tumor necrosis factor α (*Tnfa*), interferon gamma (*Ifng*), interleukin 1b (*Il1b*), *Il6*, *Il10*, *Il12b, Il13, Il15* and *Il18* that are normally expressed in the placenta^14,15,37–39^. Some of these may suppress inflammation (*Il10* and *Il13*) or are elevated with placental infection and/or inflammation (*Tnfa, Ifng, Il1, Il6*, *Il18)*^14,15,39^. Beta-actin *(Actb*) was used as the reference gene*.* Gene expression was measured by RT-qPCR*.* Statistical analysis of gene expression was performed on 2^(−ΔCq)^ values^40^. *Tnfa* was not detected in either control or infected SD placentas. *Il10* was not detected in SD placentas from infected animals (Fig. 5a). Infection in SD dams produced a significant increase in *Il15* expression with no significant change in other cytokine/chemokine genes (Fig. 5a). In WIS dams, infection induced an increase in *Ifng expression* (*P* < 0.03, Fig. 5b) with no significant change in other cytokine/chemokine genes. Collectively, the observed lack of a significant inflammatory response to infection, defined as leukocytic infiltration or enhanced expression of pro-inflammatory cytokines, suggests placentitis is not a significant contributing factor to FGR in WIS rats.

To get a visual representation of how each rat strain responded to infection, we used the mean 2^(−ΔCq)^ of the control and infected groups (n = 6) to calculate the fold change of each gene (Fig. 5c). Infected SD placentas had a twofold or greater increase in *Il6 and Il15,* whereas infected WIS placenta had a threefold decrease in *Il6* with a twofold or greater increase in *Ifng, Il13, and Il15.* Moreover, WIS placenta had detectable *Tnfa* and *Il10* that was not detectable in control and/or infected SD rats. Taken together, there were marked differences in how each rat strain responded to placental infection with *P. gingivalis*.

### Enhanced production of placental high temperature requirement A1 (Htra1) is a feature of *Pg-*induced FGR

We next evaluated the placenta for alterations in the expression of *Htra1*, follistatin like 3 protein (*Fstl3*), *Tgfb1*, activin (*Inhba*), and inhibin (*Inha*), since perturbations in their expression has been described in pre-eclamptic women with and without FGR^27,28,41,42^. Expression of these genes was measured by RT-qPCR with beta actin (*Actb*) used as the reference gene and the relative quantity 2^(−ΔCq)^^40^ values were used for statistical analysis (Fig. 5c). Infection did not alter the expression of any of these genes in the SD rat. In contrast, *P. gingivalis* infection in WIS placenta resulted in a significant increase in *Htra1* and *Fstl3* expression (*P* < 0.006 and *P* < 0.02, respectively). The overall profile of these genes in both SD and WIS rats was evaluated by measuring their fold change in response to infection (Fig. 5d). The gene expression profile in SD rats showed a decrease in the expression of *Tgfb1*, *Inha*, and *Fstl3*. In contrast, WIS placenta responded to infection with an increase in *Htra1*, *Tgfb1*, and *Fstl3.* Moreover, while a decrease in *Inha* was also observed in WIS placenta, the decrease in expression relative to control was less than half of that observed in SD rats, indicating less inhibition of *Inha* in the WIS rat strain*.*

**Figure 6:**
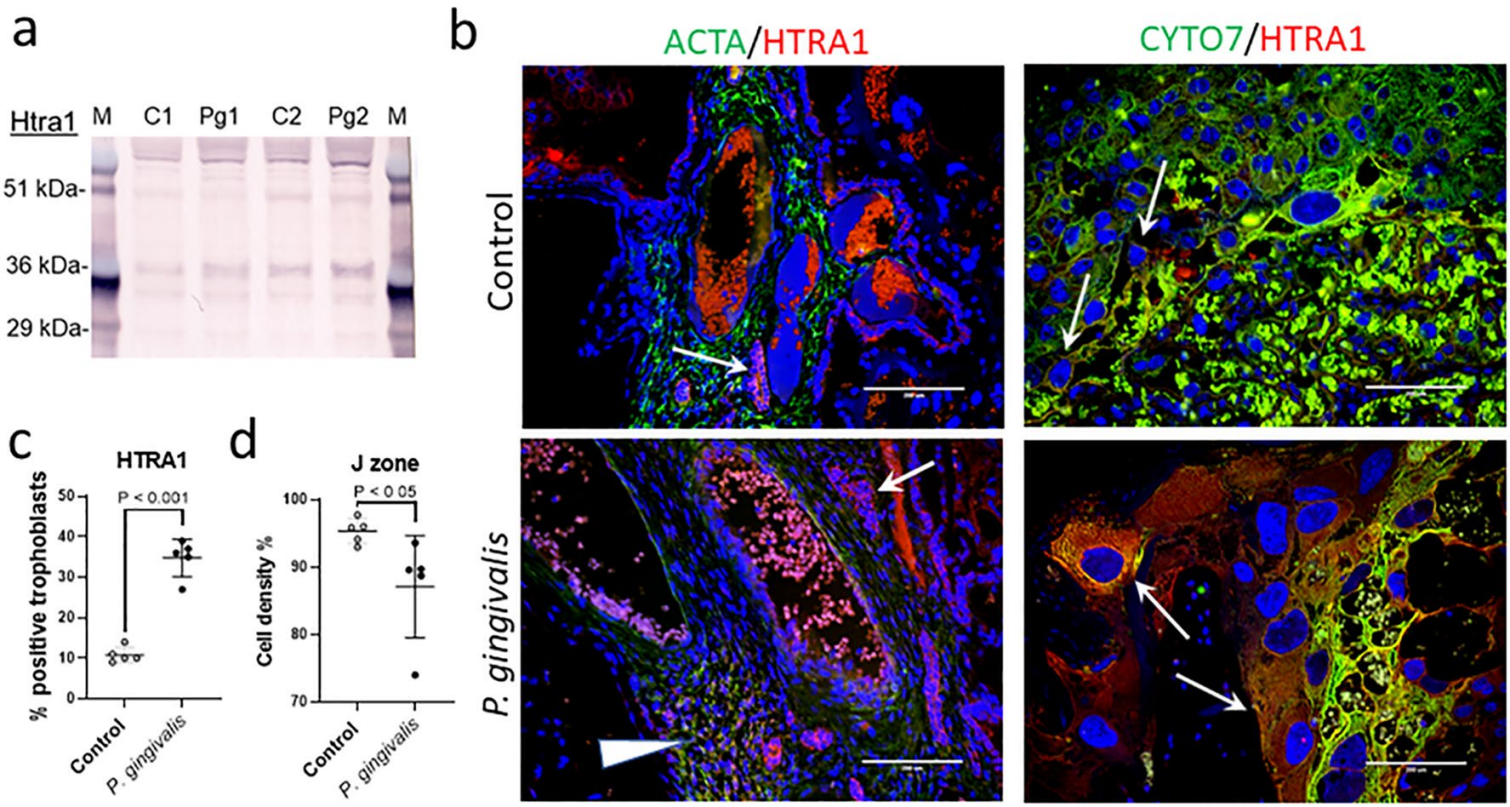
Increased placental Htra1 protein in infected WIS placenta coincides with reduced junctional zone cell density. (**a**) Cropped Western blot of Htra1 from protein extracts obtained from the same placental specimens that were used for gene expression analysis (n = 2). Full blot images and image acquisition details are available in Supplement file Fig. S7. M = SeeBlue Plus 2 pre-stained protein standard (Thermo Fisher, Waltham, MA), C = control, and Pg = *P. gingivalis*. (**b**) In situ distribution of Htra1 (red) at the chorionic plate (left panels) and the junctional zone (right panels). Antibody to α-actin (ACTA) was used to detect smooth muscle cells (green), and an antibody to cytokeratin 7 (CYTO7) was used to detect trophoblasts (green). Nuclei were stained with DAPI (blue). Arrowhead indicates HTRA1 positive smooth muscle cells. Arrows indicated HTRA1 positive trophoblasts. Scale bars = 200 µm. (**c**) Percent spongiotrophoblasts and trophoblast giant cells positive for HTRA1 staining within the junctional zone. (**d**) Morphometric assessment of cell density within the junctional zone (J zone) performed on H and E stained specimens. Horizontal bars = mean ± SD. Data analyzed by Student’s t test. Antibody details for Western blot and in situ staining are available in Supplement file Table S1. Composite image was created with Adobe Photoshop 2020 v. 21.1.3 (www.adobe.com).

To validate our findings at the protein level, Western blotting for Htra1 and FSTL3 were performed on WIS protein extracts from the same placental specimens that were used for gene expression analysis. We found no difference in FSTL3 levels between control and infected tissues (Supplement File, Fig. S7). However, when compared to controls, the 36 kDa size fragment of Htra1 that is found primarily in the cytosol^43^, was more prominent in *P. gingivalis* infected tissues than the corresponding full length 51 kDa size, which is secreted into the extracellular space^43^ (Fig. 6a). In situ staining for HTRA1 in placental specimens from SD and WIS dams was also performed. Both control and infected tissues showed HTRA1 staining in the cytosol of fibroblasts and trophoblasts lining the chorionic plate (Fig. 6b and supplement file Fig. S8). But only placenta from *P. gingivalis* infected WIS dams had more HTRA1 positive trophoblast giant cells and spongiotrophoblasts within the junctional zone (*P* < 0.001, Fig. 6c and Supplement file, Fig. S8). Since dysregulation of Htra1 is implicated in placental malformations^44^, we performed morphometry of the placental junctional zone on the same H&E stained specimens that were used for spiral arterial lumen measurements (Fig. 1). The percent trophoblast density within the junctional zone of infected WIS placenta was significantly less than controls (Fig. 6d, *P* < 0.05). On the other hand, infection did not alter trophoblast cell density in the junctional zone of control and infected SD rats (*P* = 0.7351, Supplement file, Fig. S8).

## Discussion

Intrauterine infection with *P. gingivalis* is linked to a seemingly disparate array of obstetrical syndromes such as spontaneous abortion^2^, preterm labor^3,4^, and preeclampsia with or without fetal growth restriction (FGR)^5–8^. Using a rodent model of infection, we previously demonstrated that *P. gingivalis* disrupts the physiologic remodeling of the uterine spiral arteries during pregnancy^11^, providing a common mechanistic model for how this microbe could contribute to a wide array of pregnancy disorders. One unexpected outcome from that study was the lack of FGR with *P. gingivalis* infection^11^ even though FGR can be an outcome of infection^10,13–15,45^ as well as a complication of ISAR^34^. Given that different rat strains show varying susceptibility to infection and injury^46–48^, we originally postulated that *P. gingivalis*-induced FGR was a rat strain dependent phenomenon. To test our hypothesis, we utilized a *P. gingivalis* strain that induces ISAR with a periodontitis model of infection^12^. In this study, we confirmed that ISAR was a microbial-specific phenomenon, but only WIS dams infected with *P. gingivalis* developed FGR. A unique feature of our study was that placental inflammation was not tied to FGR as reported by others^13–15^.

Unlike previous studies that saw FGR during infection with *P. gingivalis* strains W83 and A7436^13,14^, placental inflammation was not associated with FGR in this study. Tissue pathology, when detected in placental specimens from infected animals, was mild and similar as in sham controls. Although we detected increased expression of *Ifng* in infected WIS placenta, other cytokines and chemokines reported to be increased by others^13–15^ remained unchanged. The overall lack of inflammation in our model may be due to the low microbial load that we detected within these specimens that may be related to our mode of infection. For example, a high rate of placental infection with moderate placentitis can be achieved with an intravenous inoculation of *P. gingivalis* A7436 in pregnant SD rats^49^. However, an oral inoculation protocol produces a low level of infection in placental tissues and similar mesometrial pathology as what we saw in this study^11^.

There are two proposed routes whereby oral bacteria could reach the maternal–fetal interface and promote adverse pregnancy outcomes: via hematogenous dissemination from oral cavity, or by an ascending route via the genitourinary tract^50^. We readily detected *P. gingivalis* in the mesometrial triangle and junctional zone of the placenta but not within the amniotic cavity nor the chorioamnion. This pattern of bacterial colonization is suggestive of hematogenous delivery to the maternal fetal interface since the junctional zone is the first layer of the placenta to contact maternal blood. In contrast, the chorioamnion is the first tissue to be colonized by ascending bacteria from the urogenital tract^51^.

*P. gingivalis* did not induce significant changes in placental cytokine and chemokine expression in either rat strain. Nevertheless, we detected differences in the way each rat strain responded to infection (Fig. 5b). Compared to SD rats, infected WIS rats had an overall increase in the expression of *Tnf, Il13,* and *Il18*, and suppression of *Il6*. On the other hand, infected SD rats responded with an increase in *Il6*, and suppression of *Il13* and *Il18*. Due to the similar microbial load and in situ distribution of bacteria within the mesometrium and placenta of SD and WIS rats, these differences probably reflect immune responses unique to each rat strain.

Another subtle but notable difference between SD and WIS rats was the type of degeneration found in both control and infected placenta. For example, fibrinoid deposition was a feature in SD placentas that was not detected in WIS placental specimens. Fibrinoid deposition is a by-product of maternal blood-clotting that may facilitate adaptation to maternal blood flow conditions within the placental labyrinth^52^. On the other hand, degenerative features unique to WIS placenta included placental infarct, hemorrhage, and necrosis of the basal plate. In humans, placental thromboses and infarcts are the most commonly found lesions in pregnancies complicated by FGR with or without preeclampsia^53^.

Upregulation in maternal systemic inflammation is thought to be a contributing factor in FGR^54^. Experimental infection in rodents with various strains of *P. gingivalis* have been shown to increase maternal serum levels of TNF, IFN-γ, and IL-1β along with FGR^10,13,15^, which we did not see in our model. This may be due to the approach used to infect animals. For example, we used a periodontitis model that is established by oral lavage, whereas most of these studies used a subcutaneous inoculation method to infect dams^13,15^. Though the Liang et al. study also used a periodontitis model, they preceded *P. gingivalis* inoculation with a ligature placement around the molars^10^. The mechanical damage caused by the ligature, combined with *P. gingivalis* may have exacerbated periodontitis to the extent that periodontal cytokines leaked into the maternal circulation^50^.

Although our model did not produce a significant maternal inflammatory response, the repeated oral inoculations that we used to induce periodontal disease have the potential to alter maternal metabolism. *Porphyromonas gingivalis*-induced chronic periodontitis in mice alters glucose and arginine metabolism, and increases oxidative stress in their liver, brain, and heart^55^. In addition to induction of periodontal disease, oral administration of *P. gingivalis* induces gut dysbiosis in mice^56,57^ with alterations in gut metabolites that leak into the circulation and are implicated in enhancing risk for cardiovascular disease and type 2 diabetes^56^. Our inoculation protocol used *P. gingivalis* strain A7UF, which induces periodontitis in multiple rat strains^12,23^ including SD and WIS rats (supplement file, Fig. S9). Oral inoculation with *P. gingivalis* strain A7UF also changes the composition of the oral microbial community structure that remains perturbed during pregnancy^23^. Therefore, it is possible that periodontal disease along with dysbiosis is promoting maternal oxidative stress, which may be contributing to the lesions we see at the maternal–fetal interface including FGR.

HtrA1 is a serine protease that is involved in cell growth and differentiation, apoptosis, and cell senescence^24^. Disturbances in Htra1 regulation and function are implicated in vascular diseases, preeclampsia, and FGR^27,28,41,58,59^. Oxidative stress induces cellular expression of Htra1 and the autocatalytic processing of the protein into smaller, 36kDA and 29kDA proteolytically active fragments^24,43,60^. The 36 kDa fragment of Htra1 is found within the cytosol of cells, whereas the 29 kDA fragment is found within cell nucleus^43^. The proteolytic function of Htra1 degrades aberrant/denatured proteins and is thought to support cellular homeostasis^25^. In this study, placental *Htra1* gene expression along with the 36 kDa fragment of the protein were increased in infected WIS animals. Consistent with the increased concentration of the 36 kDa fragment, was the intense cytosolic staining for Htra1 protein in placental cells. Another unique feature in the WIS infected group was the greater proportion of Htra1 positive trophoblast giant cells and spongiotrophoblasts within the junctional zone of the placenta. This pattern of expression is unlikely to be normal since Htra1 expression within the junctional zone layer of the murine placenta drops dramatically after gestation day 10.5^61^, and we saw low levels of expression in control WIS rats.

The cellular pattern of overexpression of Htra1 in infected WIS placenta is consistent with cellular oxidative stress involving misfolded proteins^36,49,51^. However, the underlying mechanisms causing this dysfunction are unknown. While it is plausible that chronic periodontitis (> 3 months duration)^55^ and late gestation placental hypoxia caused by ISAR^26^ could be contributing to oxidative stress, both risk factors were also present in infected SD rats. Given that microbial loads were similar between rat strains, but host responses to infection and vascular injury were different, it is reasonable to consider that other factors are at play in the pathogenesis of FGR. For example, genetic polymorphisms that affect host response to injury^62^, or rat strain specific differences in the composition of their commensal microbial flora that may affect reproduction^63^ and we were unable to resolve with cohabitation.

While optimal levels of Htra1 support cellular homeostasis, its overexpression has detrimental effects on cell function and viability. In endothelial cells, Htra1 overexpression inhibits their proliferation, migration, and tube formation^58^, cellular processes necessary for vascular remodeling and repair. Htra1 overexpression has also been shown to promote cell senescence in embryonic fibroblasts^24^. Hypoxia-induced overexpression of Htra1 in HTR8 cells (an immortalized human EVT cell line) attenuates their ability to migrate^59^, a critical function for spiral artery remodeling and adequate placentation^64^. Transduced overexpression of Htra1 in HTR8 cells also inhibits their proliferation^59^. In this study we noted that Htra1 overexpression in WIS placental trophoblast giant cells and spongiotrophoblasts coincided with a loss of these cells within the junctional zone. Additional studies are needed to determine the cause of Htra1 overexpression and what role, if any, it has in the pathogenesis of FGR. Given the association between dysregulation in placental Htra1 in preeclampsia and FGR^27,28^, such studies are warranted.

In conclusion, using a periodontitis model of *P. gingivalis* infection, both rat strains developed ISAR indicating this is a microbe specific phenomenon. However, only infected WIS dams exhibited FGR despite both SD and WIS rats having equivalent microbial loads within the placenta. In contrast to other studies^10,15^, neither maternal systemic inflammation nor placental (fetal) inflammation was a feature of FGR in our study. However, we found structural changes within the junctional zone of the placenta of infected WIS rats that was not present in SD placentas. Furthermore, a higher proportion of junctional zone trophoblast cells in infected WIS rats were positive for cytoplasmic high temperature requirement A1 (Htra1). Cytoplasmic expression of Htra1 is a marker of cellular oxidative stress^24,25^, and placental oxidative stress secondary to tissue hypoxia is a feature of growth restricted placentas^26^. Our results show a novel phenomenon whereby *P. gingivalis* promotes FGR, which is relevant to human disease since dysregulation of placental Htra1 expression occurs in preeclamptic placentas with or without FGR^27,28^.

## Methods

### Handling and infection of rats

All experimental protocols were approved by the University of Wisconsin Institutional Animal Care and Use Committee (#V005576). All methods were carried out in accordance with relevant guidelines and regulations set forth by University of Wisconsin Institutional Animal Care and Use Committee and OLAW of the National Institutes of Health. Specific pathogen free Sprague Dawley (SD) and Wistar (WIS) rats (Charles River International Laboratories, Inc., Kingston, NY) were housed under biosafety level 2 housing within the same room. Animals were maintained under 12-h light cycles and fed sterile food and water adlib. SD and WIS rats were comingled beginning two weeks before inoculation and throughout the study so that any differences that may be present in their microbiome could be equalized between both rat strains. Animals were weighed before inoculation, after the end of the inoculation period and at time of necropsy. Control animals were always handled before infected animals to prevent cross contamination.

Periodontitis was induced as previously described^11,23^. Animals received sterile 2% carboxymethyl cellulose (CMC) or 1 × 10^9^ CFU of *P. gingivalis* strain A7UF suspended in 2% CMC administered orally for 4 consecutive days, on alternating weeks over a 12-week period (Supplement file, Fig. S9). The CFU of each inoculate was confirmed by culture. Periodontitis was confirmed by alveolar bone loss (Supplement file, Fig. S9).

Breeding was performed as previously described^11^, which was confirmed by the presence of sperm within vaginal lavage fluid. The day of breeding was deemed gestation day (GD) 0. Pregnancy was confirmed by palpation at GD 10 or 11. Dams underwent no more than two breeding cycles to be included in the study.

### Cultivation of *P. gingivalis* and preparation of oral inoculates

All inoculates of *P. gingivalis* strain A7UF were prepared from the same working stock^23^. Bacteria were passaged twice on blood agar plates supplemented with 5 µg of hemin and 1 µg vitamin K1 mL^−1^. For inoculation purposes bacteria were grown to stationary phase in tryptic soy broth supplemented with the same concentration of hemin and vitamin K. Colony forming units (CFU) were estimated by optical density readings taken at 550 nm. Broth cultures were pelleted by centrifugation at 12,000×*g* for 4 min at room temperature and resuspended in sterile 2% (w/v) carboxymethylcellulose (CMC) prepared with sterile PBS. The CFU of each inoculate was confirmed by culture.

### Necropsy and tissue processing

Pregnant dams were euthanized and necropsied at GD18. At time of necropsy, dam blood was collected for cytokine/chemokine analysis. The entire uterus was exteriorized, and 2–3 utero-placental fetal units were removed and fixed *in toto* in 10% buffered formalin to preserve the spiral arterial lumen architecture for spiral artery lumen measurements. The remaining uterus was then longitudinally transected on the anti-mesometrial side to reveal each feto-placental unit. The number of fetal resorptions were counted and recorded; these were considered embryonic/fetal deaths. Fetuses were removed for fetal weight measurements. Utero-placental units were randomly assigned to histologic evaluation or gene expression analysis. Excess uterine tissue was trimmed from the mesometrial triangle of tissues selected for histology and fixed in 10% buffered formalin overnight then washed in distilled water and transferred to 70% ethanol until processing. Placentas selected for gene expression analysis were separated from the uterus, immersed in Trizol, flash frozen in liquid nitrogen, and stored at – 80 °C until processing.

### Histology and morphometry of the mesometrial triangle and placenta

H and E stained uteroplacental specimens were coded so that the interpreter was blinded to treatment. Both halves of each section were assessed for chorioamnionitis (leukocytic infiltration of the chorioamnion, chorionic plate, and choriodecidual junction), and labyrinthitis (leukocytic infiltration of the labyrinth, the rodent equivalent of human placental villi). Semiquantitative assessments of the extent of inflammatory infiltrate into the tissue was made as follows: less than 10% was considered normal, 10–20% involvement of the specimen was considered mild; more than 20% was considered moderate to severe. Placental specimens were also evaluated for degenerative changes such as fibrinoid deposition, infarcts, hemorrhage, and coagulative necrosis at the choriodecidual interface.

Utero-placental sections that contained the maternal channel (MC) and were also positive for *P. gingivalis* antigen were used for morphometric analysis as previously described^11^. Spiral artery lumen area measurements were performed as previously described^65^ on H&E stained specimens that were fixed *in toto* since this ensured that the spiral artery architecture was not disrupted (see supplement Fig. S1). Calibrated images of each mesometrial triangle were captured with an EVOS AutoFL microscope system (Life Technologies, Grand Island, NY). The lumen of all round and ovoid spiral arterial loops within the center of the mesometrial triangle were considered the region of interest (ROI). Each ROI within each section was manually traced and the area of each loop was determined with ImageJ software (Rasband, National Institutes of Health, USA, https://imagej.nih.gov/).

VSMC retention was measured and analyzed as previously described^11,66^ using specimens that were immunostained for smooth muscle actin (ACTA). Briefly, the total circumference of each vessel loop (i.e. the ROI) was manually traced and measured with Image J software. Next, the total ACTA positive area within the ROI was also measured by tracing out the regions of the vessel that were positive for ACTA. To determine the percent VSMC, all ACTA positive area measurements within a mesometrial section were added and divided by the total area of the vessel circumference and multiplied by 100. Two mesometrial sections were analyzed from each animal (i.e. biological replicate).

EVT invasion into the mesometrial triangle was also analyzed as previously described^3, 4^ using cytokeratin 7 (CYTO7) as a trophoblast biomarker. Briefly, multiple calibrated images that spanned the entire mesometrial triangle were taken at 10× magnification with an EVOS AutoFL imaging system (Life Technologies, Grand Island, NY). The CYTO7 positive area for each calibrated image was determined with the ImageJ particle analysis feature. The final measurement of EVT invasion was generated by averaging all percent CYTO7 positive measurements that were generated for each animal.

The proportion of HTRA1 positive spongiotrophoblasts was measured on uteroplacental specimens that were immunostained for HTRA1 and CYTO7 using antibodies listed in Table S1. Multiple calibrated images of each specimen that spanned the junctional zone of the placenta were acquired at 20× magnification with an EVOS AutoFL imaging system (Life Technologies, Grand Island, NY). The proportion of HTRA1 positive spongiotrophoblasts was generated from the sum of all cell counts obtained from all images of each specimen.

Trophoblast cell density within the junctional zone was measured on the same H & E stained specimens that were used for spiral artery lumen measurements since the placental architecture was preserved by fixation. Tiled composite images that covered the entire span of the placenta were created with EVOS AutoFL imaging system and calibrated for morphometry. The junctional zone that contains spongiotrophoblasts was manually traced, and the percent cell density was determined with the particle analysis feature of Image J software 1.52v (Rasband, National Institutes of Health, USA).

### Immunofluorescent staining and imaging of the mesometrial triangle and placenta

Immunofluorescent staining was performed as previously described^11^. Antigen retrieval was performed by incubating specimens in Citrate buffer pH 6 (10 mM sodium citrate with 0.05% Tween 20) heated at 95 °C. Antigen retrieval in specimens to be stained with anti-CYTO7 antibody was performed Tris–EDTA buffer pH 9 (10 mM Tris base with 0.05% EDTA), which was performed as already described. All antibodies used in this study are summarized in Table S1.

Stained tissue sections were observed and imaged using an EVOS AutoFL microscope system (Life Technologies, Grand Island, NY). The light cubes used for fluorescent imaging were GFP, Texas Red, Cy5 and DAPI (Life Technologies, Grand Island, NY) for detecting ALEXA 488, ALEXA 594, ALEXA 647 and DAPI, respectively. Camera settings for imaging of fluorescent stains were optimized using the tissue section with the highest positive fluorescent signal. Once optimized, the settings were then kept the same while imaging all other tissue sections for a specific staining experiment.

### Isolation of RNA from Placenta

Tissue collected during necropsy was immersed in TRIzol Reagent (Life Technologies, Cat# 15596-018) at a ratio of 1 mL of reagent per 50–100 mg of tissue. Samples were stored at − 80 °C in RNAase free tubes until processing. At time of tissue processing, tissues were minced in TRIzol followed by homogenization with a Read Mill 4 homogenizer (Thermo Fisher Scientific, Waltham, MA). Extraction of total RNA was performed according to manufacturer’s instructions. The RNA pellet was resuspended in 20–50 µL of RNase-free water. Genomic DNA was removed by digestion using Turbo DNA-free Kit (Ambion AM1907) following the manufacturer’s protocol. The concentration and quality of the RNA from each sample was determined with an Agilent bioanalyzer 2100 Instrument (Agilent Technologies, Inc, Santa Clara, CA).

### Placental gene expression analysis

Conversion of total RNA to cDNA and qPCR was performed with GoTaq 2 -Step RT-qPCR System (Promega Corp, catalog #A6010, Madison, WI) using primers listed in Table S2 according to manufacturer’s instructions. RT-qPCR was performed with a LightCycler 96 Roche Real-Time PCR system (Roche Diagnostics, Indianapolis, IN). Briefly, 88 ng of cDNA was amplified by qPCR with 1 µM of each primer using the following conditions: preincubation (95C for 60S), followed by 45 amplification cycles (95C for 10 s, 55C for 10 s, 72C for 10 s), and a final cycle (95C for 5 s, 72C for 30 s, and cooling at 37C for 30 s). QPCR reactions were performed in duplicate and all genes listed in Table S2 were analyzed at the same time. Each 96 well plate included one control and one infected sample. Relative gene expression for each specimen was determined by the comparative Cq method with *Actb* used as the reference and values reported as 2^−(ΔCq)^^40^. All primer pairs listed in Table S2 have similar primer efficiencies as shown by logarithmic PCR amplification plots^40^ (Fig. S1).

### Detection of *P. gingivalis* 16 s gene and fetal sex

Placental genomic DNA was extracted with TRIzol reagent (Thermo Fisher Scientific, Waltham, MA) according to manufacturer's instruction. For improved detection of *P. gingivalis*, an aliquot of placental tissue was processed with HostZERO Microbial DNA Kit (catalog#D4310, Zymo Research, Irvine, CA). DNA concentrations were determined by absorbance measured at 260nm using a Thermo Scientific NanoDrop 2000 Lite Spectrophotometer (Thermo Fisher Scientific, Waltham, MA). The 16s gene of  *P. gingivalis* was detected with Porph16S_F:AGGCAGCTTGCCATACTGCG and Porph16S_R1:ACTGTTAGCAACTACCGATGT primer sets. Placental specimens from both sham inoculated and *P. gingivalis* infected animals were analyzed within the same assay. Each qPCR reaction used purified *P. gingivalis* genomic DNA as a positive control. Genomic DNA from a purified culture of *P. gingivalis*  was used to generate a standard curve. The level of detection of *P. gingivalis* DNA was approximately 0.01 pg. In our assay, < 37 cycles was used as the cut-off for a positive signal since none of our sham control animals were positive within this cycle range. The male *Sry1* gene was detected with SRY1-2F:TGGACATCCCCACTGGATACC and SRYR1-2R:CTATGGTGCAGGGTCGGTCA primer sets.

All qPCR reactions were performed with Platinum SuperFi Green PCR Master Mix (Thermo Fisher Scientific, Waltham, MA) using a Light Cycler 96 (Roche Applied Science, Indianapolis, IN) with the following amplification protocol: Start-up at 95 °C for 2 min followed by 40 cycles of 95 °C for 15 s, 56.7  for 15 s, and 60 °C for 60 s.

### Western blot analysis

Protein pellets were resuspended in 170 μls of Protein buffer [7 M urea, 2 M thiourea, 4% chaps, 65 mM DTT, 0.1% protease inhibitor cocktail (SIGMA, catalog #SBB-20)]. Total protein concentrations were determined by absorbance at 280 nm with a Thermo Scientific NanoDrop 2000 (Thermo Fisher, Waltham MA). Twenty-eight μg of total protein from each sample were subjected to gel electrophoresis using a Bolt 4–12% Bis–Tris Plus gel (Thermo Fisher, Waltham MA) and transferred to nitrocellulose paper with an iBlot 2 system (Thermo Fisher, Waltham MA). After blocking in TBS buffer (20 mM tris base, 137 mM NaCl, pH adjusted to 7.6 with HCl) with 3% dry milk (Cat# M084, Lab Scientific Inc., Danvers MA), and incubating at 4 °C overnight, the membranes were washed 3 times with TBS washing buffer (TBS with 0.05% Tween-20). Blocked membranes were incubated with primary antibody (Table S1 in supplement file) for 3 h at room temperature, then washed three times in TBS wash buffer. Membranes were then incubated for 1 h at room temperature with goat anti-rabbit-Alkaline Phosphatase conjugated antibody (catalog # S3731 at 1:7,500 dilution, Promega Corp., Madison, WI) then washed 2 times with TBS wash buffer followed by 1 wash with AP buffer (0.1 M Tris base, 0.1 M NaCL, 5 mM MgCL2, pH 9.5) for 10 min at room temperature. The chromogenic signal was developed with 1-Step NBT/BCIP substrate (Thermo Fisher, Waltham MA). Blots were digitized with an Epson Perfection V800 Scanner (Epson America, Inc., Hillsboro, OR). The image file was cropped, labeled and saved as a tiff file with Microsoft 365 Power Point v 2006 (Microsoft.com). Image resolution was increased to 300 dpi with Adobe Photoshop V 21.1.3 (www.Adobe.com).

### Serum cytokine profiling

Serum was collected and stored at − 80 °C until analysed. Fifty microliters of serum were analyzed for IL-1α, G-CSF, IL-10, IL-17A, IL-1β, IL-6, TNFα, IL-4, GM-CSF, IFNγ, IL-2, IL-5, IL-13, IL-12p70, Eotaxin, GRO-α, IP-10, MCP-1, MCP-3, MIP-1α, MIP-2, and Rantes (Rat Cytokine & Chemokine 22-plex ProcartaPlex Panel, Invitrogen, Catalog #EPX220-30122-901) according to manufacturer’s instructions using a Luminex 200 instrument (Thermo Fisher Scientific, Waltham MA).

#### Statistical analysis

One way ANOVA, student’s t test, and chi-square analysis was performed with GraphPad Prism version 8.4.2 Software (GraphPad Software, LLC, www.graphpad.com), with *P* < 0.05 being considered significantly different.

## Supplementary information

Supplementary information
