# Supplementary material for: Fetal growth restriction is a host specific response to infection with an impaired spiral artery remodeling-inducing strain of Porphyromonas gingivalis
Source: Sci Rep. 2020 Sep 3;10:14606. doi: 10.1038/s41598-020-71762-9 (PMC7471333; doi:10.1038/s41598-020-71762-9)
Supplement: Supplementary file 1 — Supplementary information [file 41598_2020_71762_MOESM1_ESM.pdf]

Fetal growth restriction is a host specific response to infection with an impaired spiral artery remodeling-inducing strain of *Porphyromonas gingivalis*

Tanvi Tavarna[1], Priscilla L. Phillips[2], Xiao-jun Wu[1], and Leticia Reyes[1]*

Table S1. Antibodies used for immunofluorescent staining and Western blot

Target antigen	Dilution	Host Organism	Catalog #, Manufacturer
P. gingivalis	1:2000	Rabbit polyclonal	Lot C7947, Custom made
Cytokeratin-7	1:200	Mouse (clone LP1K)	Ab20206, Abcam (Cambridge, MA)
α-actin	1:200	Mouse (clone 1A4)	MCA5781GA, Biorad Laboratories (Hercules, CA)
Htra1	1:200/1:1000	Rabbit polyclonal	PA5-79419, Thermofisher (Waltham MA)
Fstl3	1:500	Rabbit polyclonal	Orb183779, Biorbyt (San Francisco, CA)
Mouse isotype	1:200	Mouse IgG	31903, Thermofisher (Waltham MA)
Rabbit isotype	1:2000	Rabbit polyclonal	Lot C7947, pre-immune serum
Rabbit Ig ALEXA 594	1:1000	Goat	A-11037, Life Technologies (Grand Island, NY)
Mouse Ig ALEXA 594	1:1000	Goat	A-11032, Life Technologies (Grand Island, NY)
Rabbit Ig ALEXA 647	1:1000	Goat	A32733, Life Technologies (Grand Island, NY)
Mouse Ig ALEXA 647	1:1000	Goat	A32728, Life Technologies (Grand Island, NY)
Rabbit Ig ALEXA 488	1:1000	Goat	A32731, Life Technologies (Grand Island, NY)
Mouse Ig ALEXA 488	1:1000	Goat	A32723, Life Technologies (Grand Island, NY)

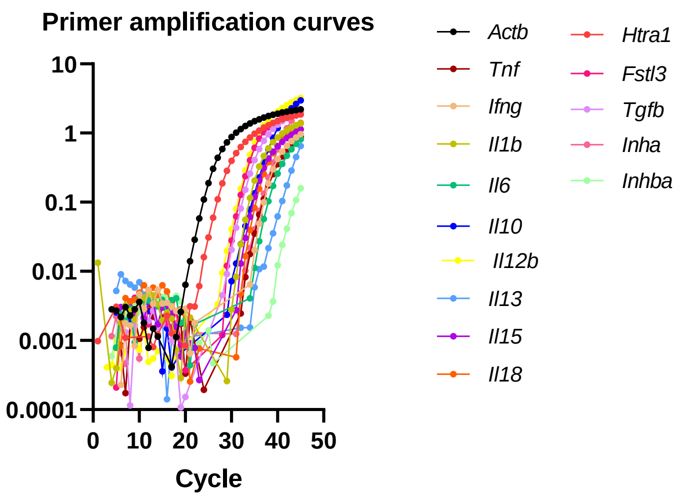

Figure S1: Amplification plots of primers listed in Table S2. Data was generated from WIS placental tissues in which all genes were detected.

Table S2. Primers used for gene expression analysis

Gene	Primer	Primer Sequence
Tnf	Qiagen QT00178717	-
Ifng (NM_138880.2)	Integrated DNA Technologies (IDT) Forward 1	CGAATCGCACCTGATCACTAA
	IDT Reverse 1	TGGATCTGTGGGTTGTTCAC
IL1b	Qiagen QT00181657	-
IL 6	Qiagen QT00182896	-
IL 10	Qiagen QT00177618	-
IL 12b	Qiagen QT00188839	-
IL 13	Qiagen QT00184842	-
IL 15	Qiagen QT01813637	-
IL 18	Qiagen QT00183071	-
Htra 1	Qiagen QT00178017	-
Tgfb1	Qiagen QT00187796	-
Inha	Qiagen QT00370258	-
Inhba	Qiagen QT00183918	-
Fstl3	Qiagen QT00191044	-
Actb	Qiagen QT00193473	-

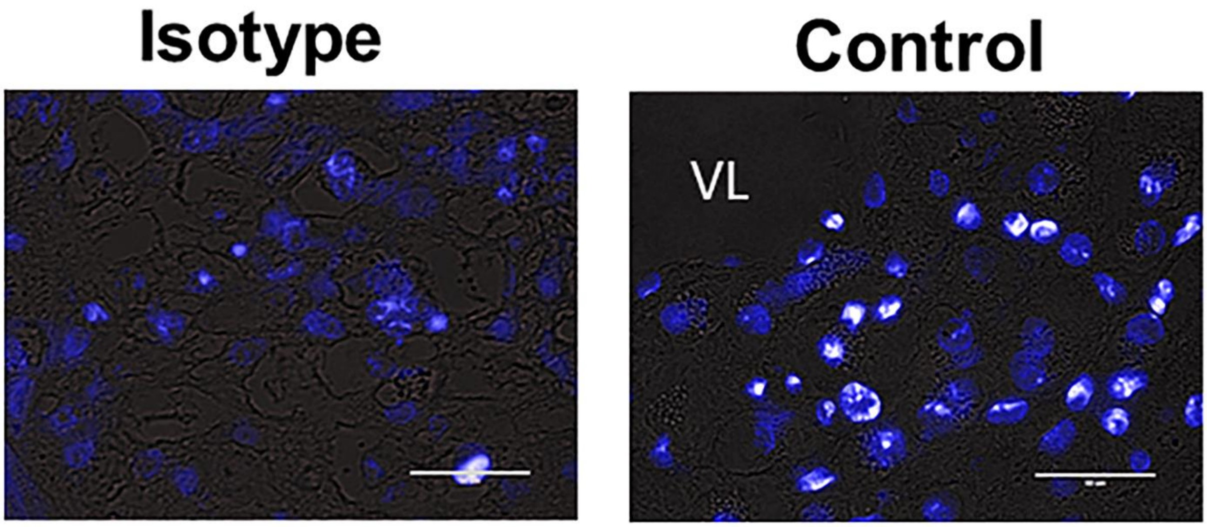

Figure S2. Staining controls for *in situ* detection of *P. gingivalis* proteins in uteroplacental tissues. Transillumination (grey) was used to define the tissue architecture. VL = vessel lumen. Nuclei were stained with DAPI (blue). Scale bar = 50 µm.

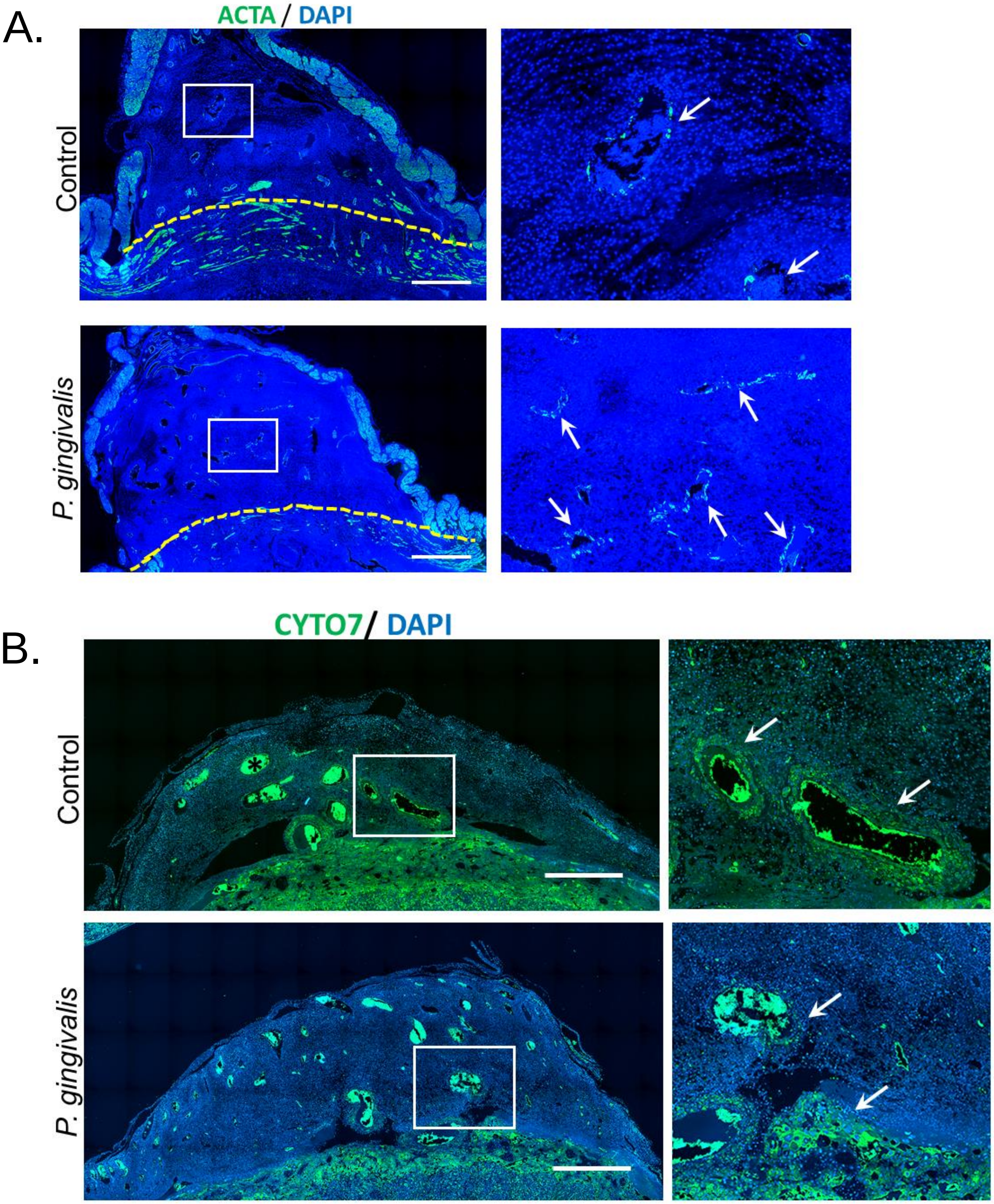

Figure S3. Representative images of immunostained vascular smooth muscle cells (VSMC) (A) and extravillous trophoblasts (B) in GD18 placentae. Images are tiled composites of individual pictures taken at 10X magnification with an EVOS Auto FL imaging system. A) Vascular smooth muscle cells (VSMC) were identified by α-actin (ACTA) staining. Yellow dash line demarcates the edge between the decidua and the mesometrial triangle. B) Extravillous trophoblasts (EVT) were identified by cytokeratin 7 (CYTO7) staining. Boxed regions demarcate the magnified region shown in the corresponding panel on the right. White arrows indicate regions of interest (retained VSMC or invading EVT) Scale bar = 1000 µm.

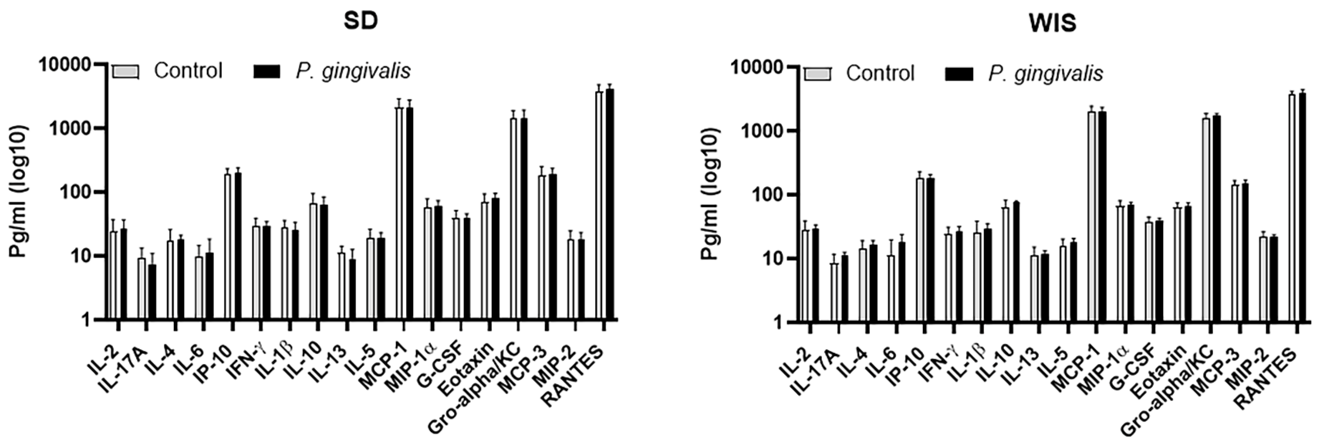

Figure S4. Detection of circulation cytokines and chemokines in maternal serum (n = 5). Fifty microliters of rat serum from each animal was used for analysis. Cytokines and chemokines were measured with a multiplex immunoassay (Rat Cytokine & Chemokine 22-plex ProcartaPlex Panel, Invitrogen, Catalog #EPX220-30122-901). The assay works on the principles of a sandwich ELISA on a magnetic bead platform and protein targets were measured with a Luminex instrument (ThermoFisher Scientific, Waltham MA).

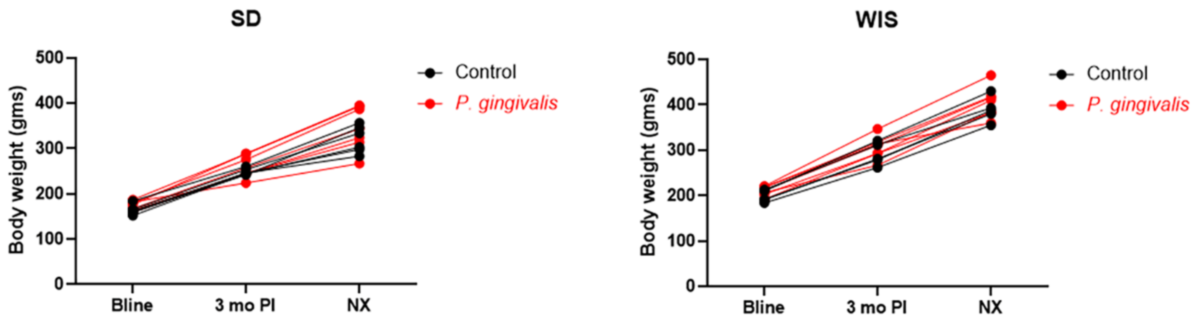

Figure S5. Maternal weight gain from pre-inoculation (Bline), to end of inoculation phase (3 mo PI) to time of necropsy (NX) at GD18. Statistical analysis was performed on the slope of the line that was used to measure the degree of weight gain of each animal. One-way ANOVA of the slope of the lines indicated that there was no difference in weight gain among the groups (P = 0.2347). Intrastrain student's t tests also indicated that infection did not affect the rate of maternal weight gain (P = 0.4531 for SD, and P = 0.7398 for WIS).

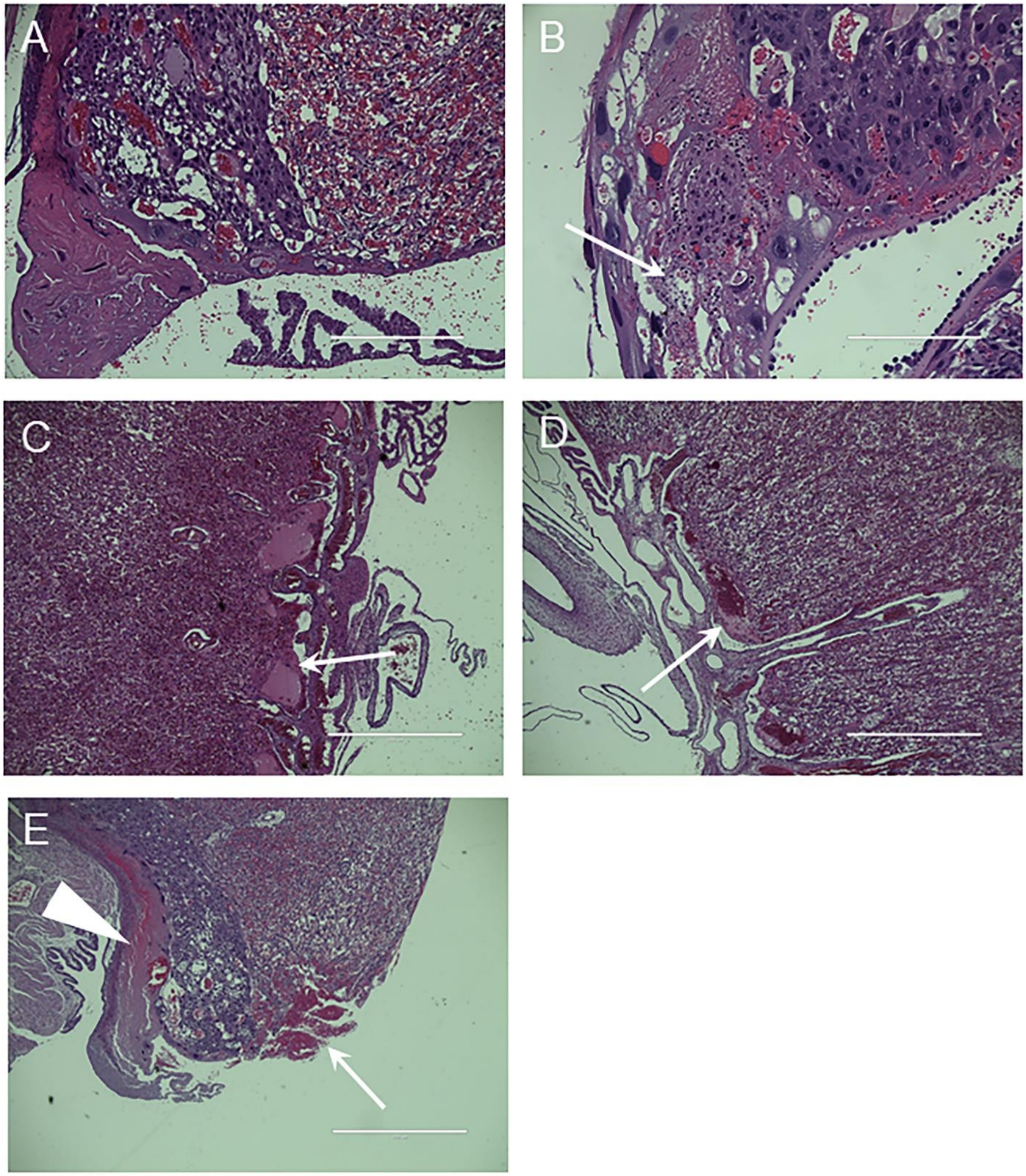

Figure S6. Images of GD18 SD and WIS placenta. A) Choriodecidual junction from a control specimen with no pathology. B) Leukocytic infiltration of the choriodecidual junction (white arrow**)** representative of a specimen with mild inflammation. C) A placental specimen with fibrinoid deposits near the chorionic plate (arrow). D) A placental specimen with placental infarcts near the chorionic plate (arrow). E) A placental specimen with a band of coagulative necrosis at the basal plate (arrowhead) and hemorrhage (arrow). Scale bar = 1000 µm.

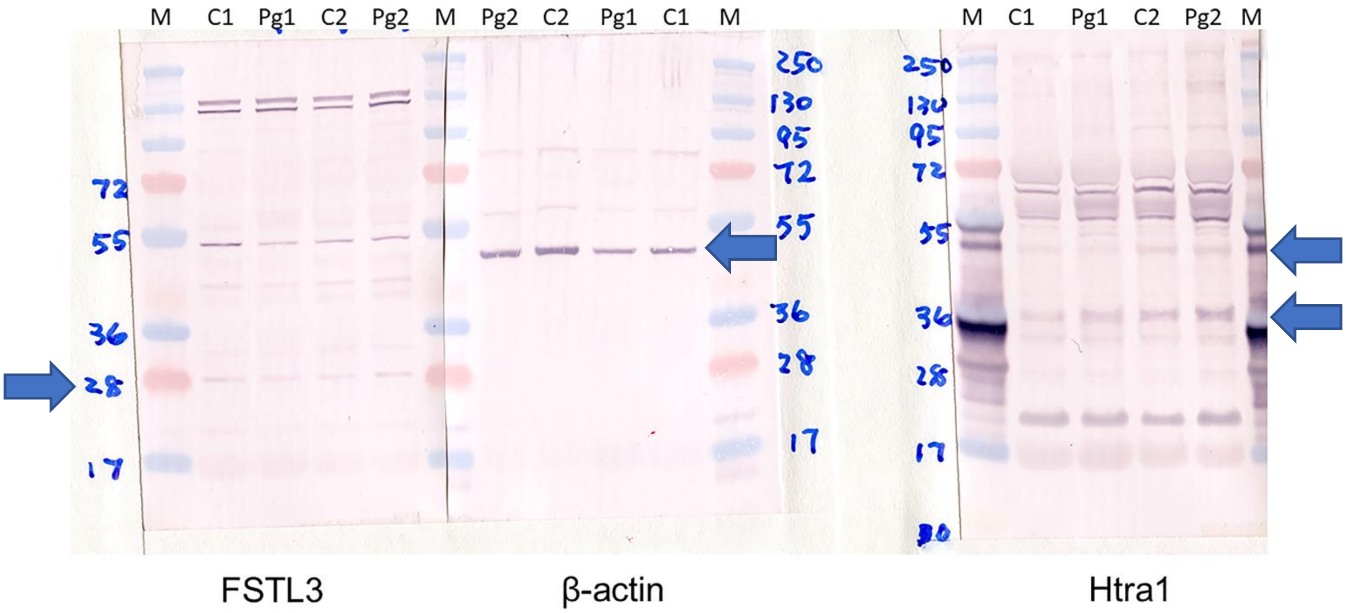

Figure S7. Western blots of Follistatin-related protein 3 (FSTL3), β-actin, and Htra1 protein extracts obtained from the same placental specimens that were used for gene expression analysis (n = 2). All blots are from the same protein extracts. FSTL3 (28 kDa) and β-actin (42 kDa) were run on the same gel and transferred to the same blot but were separated for final immunostaining. Protein extracts for detection of Htra1 were run on a separate gel. To obtain a digital image, all blots were simultaneously scanned with an Epson Perfection® V300 scanner. The digital image was imported into Microsoft Office 365 Power Point® for labeling columns and saved as a tiff file that was imported into Adobe Photoshop V 21.1.3 to increase picture resolution to 300 dpi. M = SeeBlue™ Plus2 pre-stained protein standard (Thermofisher, Waltham, MA), C = control, and Pg = *P. gingivalis.* Blue arrows indicate the bands of interest.

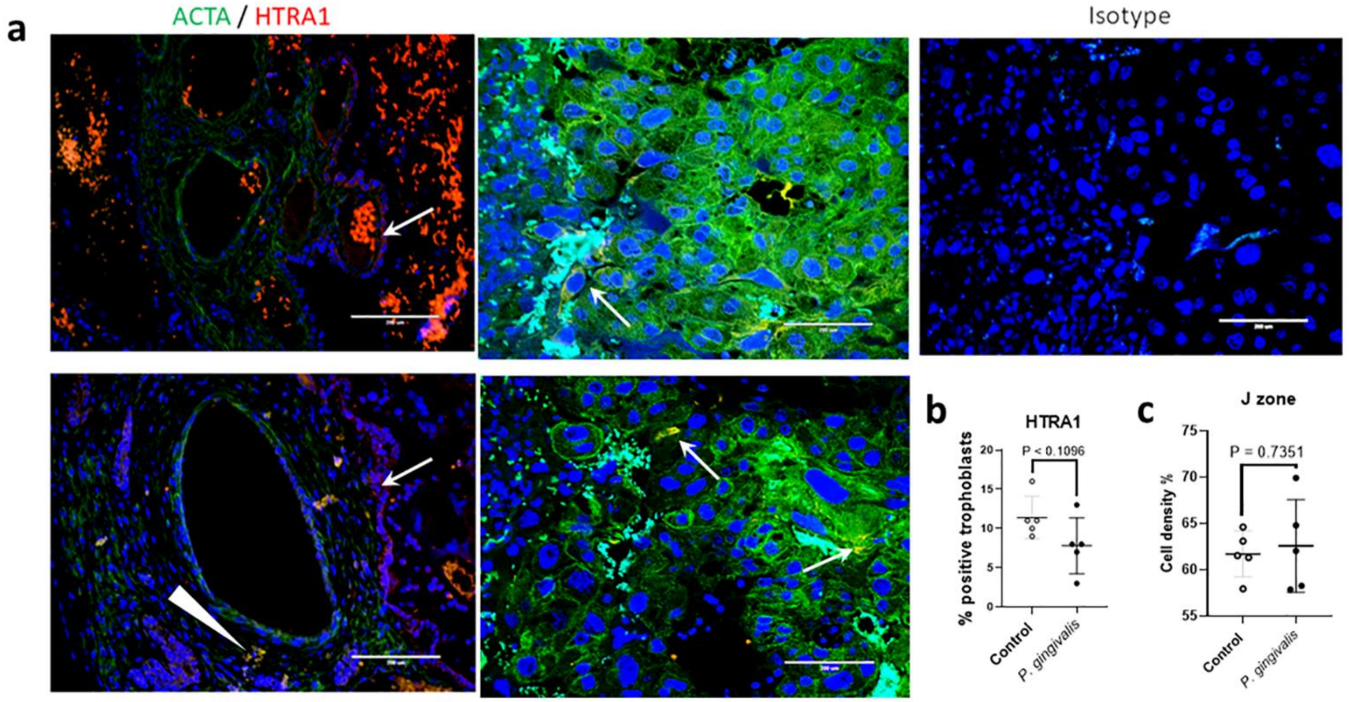

Figure S8. Htra1 staining in SD placenta and spongiotrophoblast density. (A) *In situ* distribution of Htra1 (red) the chorionic plate (left panel) and at the junctional zone (middle panel) of control and *P. gingivalis* infected SD placenta. Antibody to α-actin (ACTA) was used to detect smooth muscle cells (green), and an antibody to cytokeratin 7 (CYTO7) was used to detect trophoblasts (green). Nuclei were stained with DAPI (blue). Arrowhead indicates HTRA1 positive smooth muscle cells. Arrows indicate HTRA1 positive trophoblasts. Scale bars = 200 µm. **c** Percent spongiotrophoblasts and trophoblast giant cells positive for HTRA1 staining within the junctional zone. **d** Morphometric assessment of cell density within the junctional zone (J zone) performed on H and E stained specimens. Horizontal bars = mean ± SD. Data analyzed by Student’s t test.

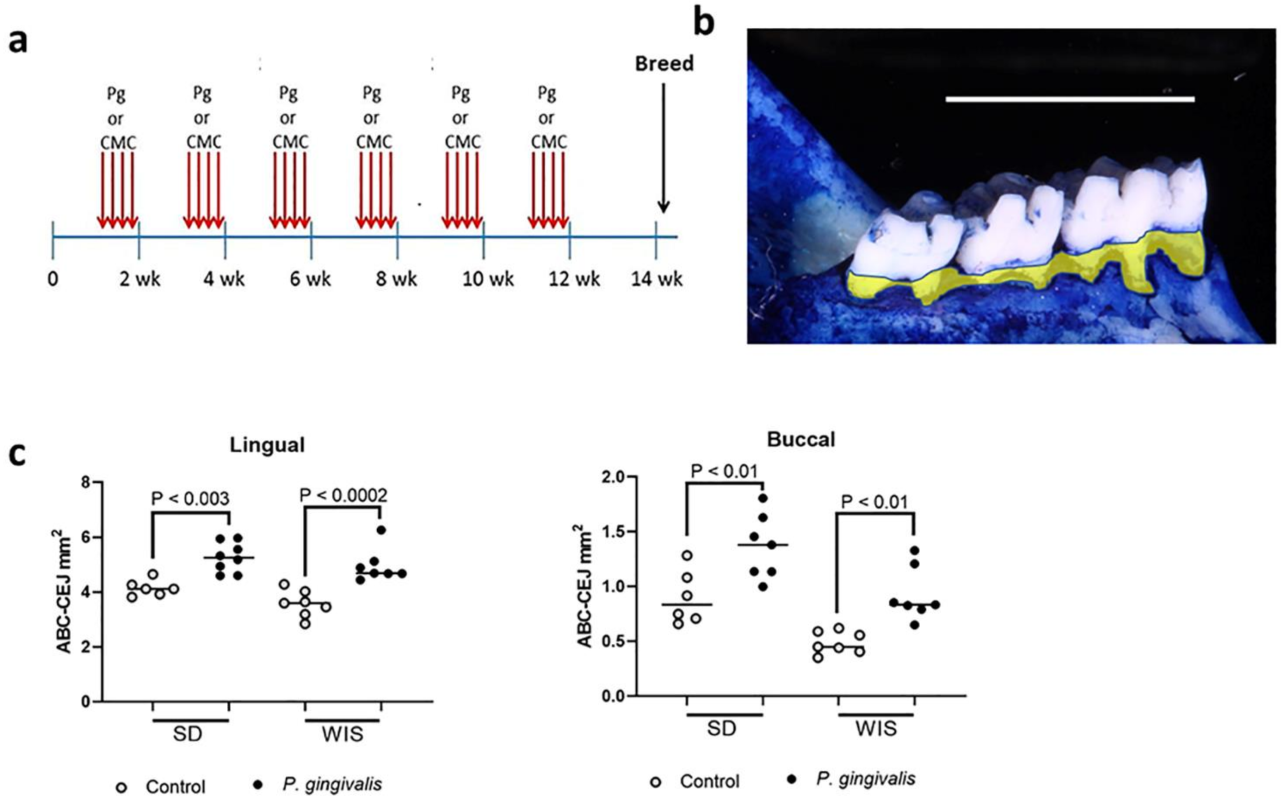

Figure S9. Periodontitis model used in this study. (A) Oral inoculation scheme used to produce periodontitis. (B) A representative image of the lingual side of the mandible. The yellow region illustrates how alveolar bone loss was traced and measured, which extends from the cemento-enamel junction (CEJ) to the alveolar bone crest (ABC). Scale bar = 5 mm. (C) The extent of alveolar bone loss in both SD and WIS rats.
